# A Low-Cost, Ear-Contactless Electronic Stethoscope Powered by Raspberry Pi for Auscultation of Patients With COVID-19: Prototype Development and Feasibility Study

**DOI:** 10.2196/22753

**Published:** 2021-01-19

**Authors:** Chuan Yang, Wei Zhang, Zhixuan Pang, Jing Zhang, Deling Zou, Xinzhong Zhang, Sicong Guo, Jiye Wan, Ke Wang, Wenyue Pang

**Affiliations:** 1 Department of Cardiology Shengjing Hospital of China Medical University Shenyang China; 2 Department of Pulmonary and Critical Care Medicine Shengjing Hospital of China Medical University Shenyang China; 3 Sewickley Academy Senior High School Pittsburgh, PA United States; 4 School of Population Health University of New South Wales Sydney Australia; 5 Department of Cardiac Surgery Shengjing Hospital of China Medical University Shenyang China

**Keywords:** stethoscope, auscultation, COVID-19, Raspberry Pi, Python, ear-contactless, low-cost, phonocardiogram, digital health

## Abstract

**Background:**

Chest examination by auscultation is essential in patients with COVID-19, especially those with poor respiratory conditions, such as severe pneumonia and respiratory dysfunction, and intensive cases who are intubated and whose breathing is assisted with a ventilator. However, proper auscultation of these patients is difficult when medical workers wear personal protective equipment and when it is necessary to minimize contact with patients.

**Objective:**

The objective of our study was to design and develop a low-cost electronic stethoscope enabling ear-contactless auscultation and digital storage of data for further analysis. The clinical feasibility of our device was assessed in comparison to a standard electronic stethoscope.

**Methods:**

We developed a prototype of the ear-contactless electronic stethoscope, called Auscul Pi, powered by Raspberry Pi and Python. Our device enables real-time capture of auscultation sounds with a microspeaker instead of an earpiece, and it can store data files for later analysis. We assessed the feasibility of using this stethoscope by detecting abnormal heart and respiratory sounds from 8 patients with heart failure or structural heart diseases and from 2 healthy volunteers and by comparing the results with those from a 3M Littmann electronic stethoscope.

**Results:**

We were able to conveniently operate Auscul Pi and precisely record the patients’ auscultation sounds. Auscul Pi showed similar real-time recording and playback performance to the Littmann stethoscope. The phonocardiograms of data obtained with the two stethoscopes were consistent and could be aligned with the cardiac cycles of the corresponding electrocardiograms. Pearson correlation analysis of amplitude data from the two types of phonocardiograms showed that Auscul Pi was correlated with the Littmann stethoscope with coefficients of 0.3245-0.5570 for healthy participants (*P*<.001) and of 0.3449-0.5138 among 4 patients (*P*<.001).

**Conclusions:**

Auscul Pi can be used for auscultation in clinical practice by applying real-time ear-contactless playback followed by quantitative analysis. Auscul Pi may allow accurate auscultation when medical workers are wearing protective suits and have difficulties in examining patients with COVID-19.

**Trial Registration:**

ChiCTR.org.cn ChiCTR2000033830; http://www.chictr.org.cn/showproj.aspx?proj=54971.

## Introduction

Since the outbreak of COVID-19, increasing numbers of physicians and nurses have been treating patients on the front lines worldwide. Many health care workers have been exposed to SARS-CoV-2 at work, and some of them have become infected with the virus due to high rates of nosocomial transmission [1-5]. Many medical professionals have emphasized the importance of safety measures during the management of critical patients [5,6].

The stethoscope is a useful instrument for physicians, nurses, anesthetists, and other health professionals who examine, diagnose, and evaluate the respiratory status of patients with COVID-19. Nearly all critically ill patients with COVID-19 present severe and acute respiratory conditions [7]. Auscultation is important for these patients, particularly those with severe pneumonia or respiratory dysfunction and those who are intubated and whose breathing is assisted with a ventilator, to ensure accurate diagnosis and to assess disease severity and treatment efficacy [8,9]. In addition, auscultation has been shown to act as an emotional bridge between health staff and patients, who are isolated and separated from their loved ones [10].

**Figure 1:**
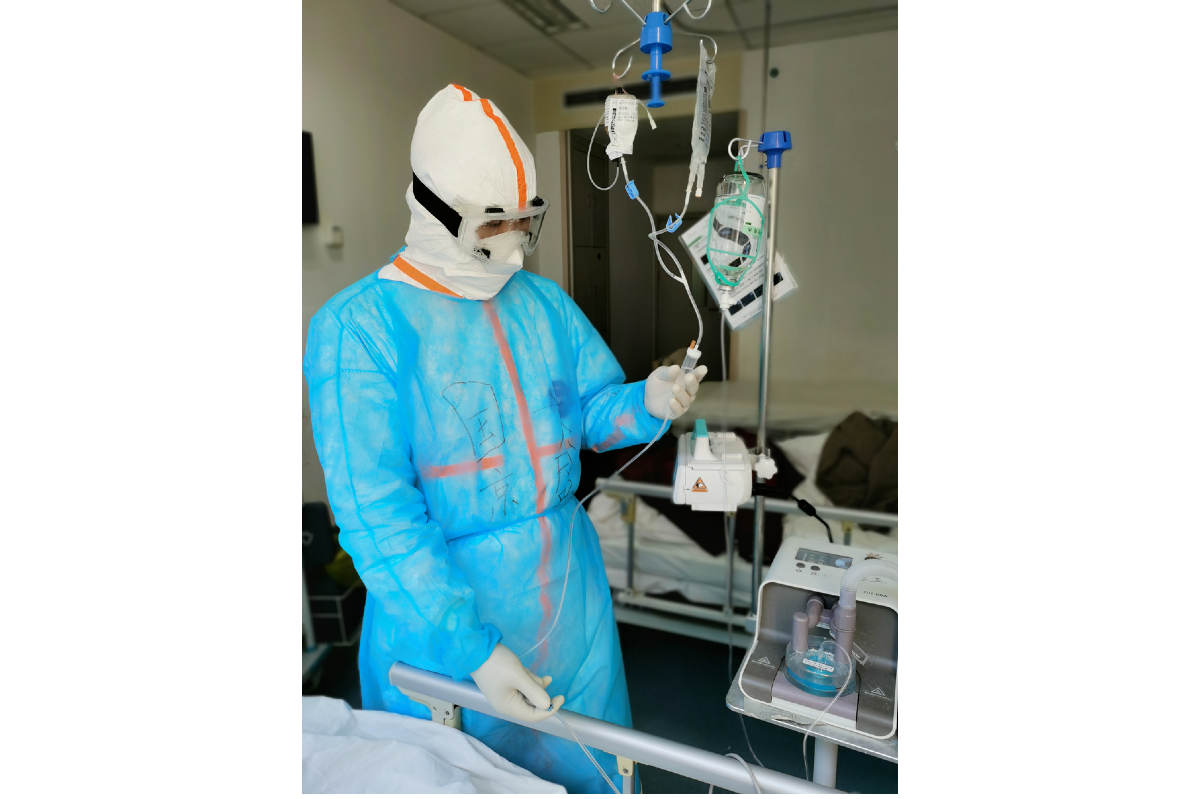
A medical staff member nursing a patient with COVID-19 in Wuhan, China. The staff member’s protective clothing prevents the use of a conventional stethoscope.

However, some researchers have found that stethoscopes can spread infection between patients and health care professionals [11,12] and that stethoscopes are not cleaned sufficiently often by medical staff [13]. As a consequence, the safety of stethoscope implementation for chest auscultation during the COVID-19 pandemic has been questioned [14]. Furthermore, inside the quarantine wards in hospitals, medical staff wearing protective clothing are unable to use conventional stethoscopes because their protective clothing covers their ears [9,15] (Figure 1). As a safer alternative, some experts have suggested using stethoscopes less frequently and ultrasound more frequently [16], while other experts have stressed the necessity of stethoscope use and auscultation in COVID-19 treatment [9].

Electronic stethoscopes can transmit auscultation sounds via Bluetooth and enable users to store and replay the sounds through a personal computer or other device. For example, the Littmann 3200 electronic stethoscope (3M) has higher sensitivity and specificity than classic acoustic stethoscopes for diagnosis of patients with heart vascular disease [17]. Although an electronic stethoscope possesses these benefits, it still requires contact listening via the ears of medical staff, which is unsafe when working with patients with COVID-19. Furthermore, electronic stethoscopes are expensive, with prices greater than US $350, which limits their use in low-resource settings. During the COVID-19 pandemic, many medical facilities encountered a critical care crisis due to their limited medical capacity, shortages of personnel [18-20], and shortages and increasing cost of health care products, including ventilators and other medical devices [21,22].

Some manufacturers and researchers have integrated stethoscopes with smartphones, such as the Eko Core Digital Stethoscope (Eko Devices) [23]. However, this type of stethoscope can only transfer the auscultation data to a smartphone, tablet, or a personal computer; therefore, real-time playback to other medical staff is difficult. Furthermore, the smartphone is inconvenient for use in an intensive care unit (ICU) for COVID-19. One proposed solution is to capture and analyze heart sounds using only a smartphone [24]. In that study, researchers recorded normal and pathological heart sounds using three different smartphones, and diagnosis was performed using machine learning. However, the device and procedure were designed for intelligent diagnosis and not for application during management of patients with COVID-19, in addition to the abovementioned difficulties of using the smartphone in an isolated ICU. Stethoscopes and devices on the market or published in the literature require ear contact, which is not feasible for staff wearing personal protective equipment.

As medical staff participating in the frontline treatment of patients with COVID-19 in Wuhan, China, our team realized the need for a stethoscope that did not require ear contact for auscultation. Here, we describe the design and development of an electronic stethoscope (Auscul Pi) based on a low-cost, single-board computer the size of a credit card (Raspberry Pi) for ear-contactless recording and archiving of auscultation results. We explored the usability and advantages of the new stethoscope in an exploratory sample of patients and healthy volunteers in comparison with the Littmann 3200 electronic stethoscope.

## Methods

### Development

#### Design

**Figure 2:**
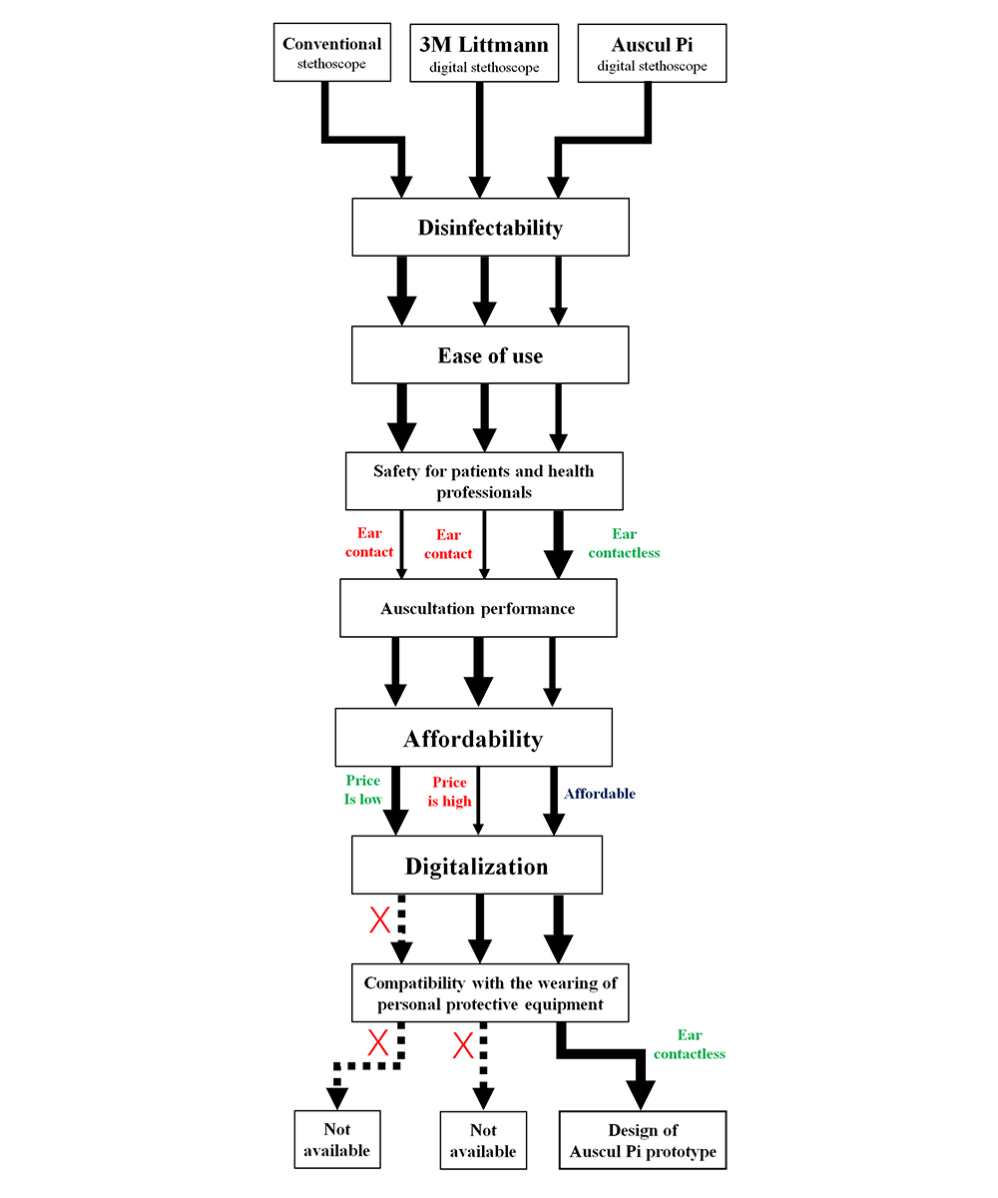
Flowchart of Auscul Pi design based on the evaluation of 7 dimensions in comparison with a conventional stethoscope and 3M Littmann digital stethoscope. Thick solid arrows indicate that the stethoscope performed satisfactorily on the indicated dimension; dashed arrows indicate that it did not.

The Auscul Pi electronic stethoscope protype was designed and developed using Raspberry Pi hardware (Raspberry Pi Foundation), the open-source Python programming language, and other modified components. We evaluated the prototype for use with patients with COVID-19 in terms of seven dimensions: disinfectability, ease of use, safety for patients and health professionals, auscultation performance, affordability, digitalization, and compatibility with the wearing of a personal protective suit. The prototype was compared with a conventional stethoscope and a Littmann digital stethoscope (Figure 2).

#### Hardware

Raspberry Pi computers were developed by the Raspberry Pi Foundation in 2009 with the original purpose of computer science education [25]. This small single-board computer (up to the size of a credit card) consists of system-on-a-chip hardware, including a quad-core ARM processor (ARM Holdings), 1 GB of memory, and a graphic processing unit. Wi-Fi, Bluetooth, Ethernet and other modules are also built into the computers. The components we used for this project, all of which are generic and can easily be purchased on the web, are listed in Table 1.

**Table 1:** Components of the Auscul Pi digital stethoscope.

Item	Model	Manufacturer	Quantity	Price in ￥ (Price in US $)^a,b^
Raspberry Pi	3 Model B+	Raspberry Pi Foundation	1	280 (39.27)
MicroSD^c^ card	64G	SanDisk	1	59 (8.27)
Micro USB power supply	N/A	MingXing	1	5.88 (0.82)
UPS^d^ battery expansion board	5-volt output	YDSM	1	132 (18.51)
18650 rechargeable batteries	INR19860-30Q 3000MA	YDSM	2	48 (6.73)
Microphone	USB collar microphone	QianBaiXiang	1	29 (4.07)
Speaker	Inserted microspeaker	Yayusi	1	56.9 (7.98)
Touch screen	3.5-inch touch screen	Mumu	1	59 (8.25)

^a^Based on an exchange rate of 7.13 RMB=US $1.

^b^Total cost: ￥669.78 (US $93.90)

^c^SD: secure digital.

^d^UPS: uninterruptible power supply.

**Figure 3:**
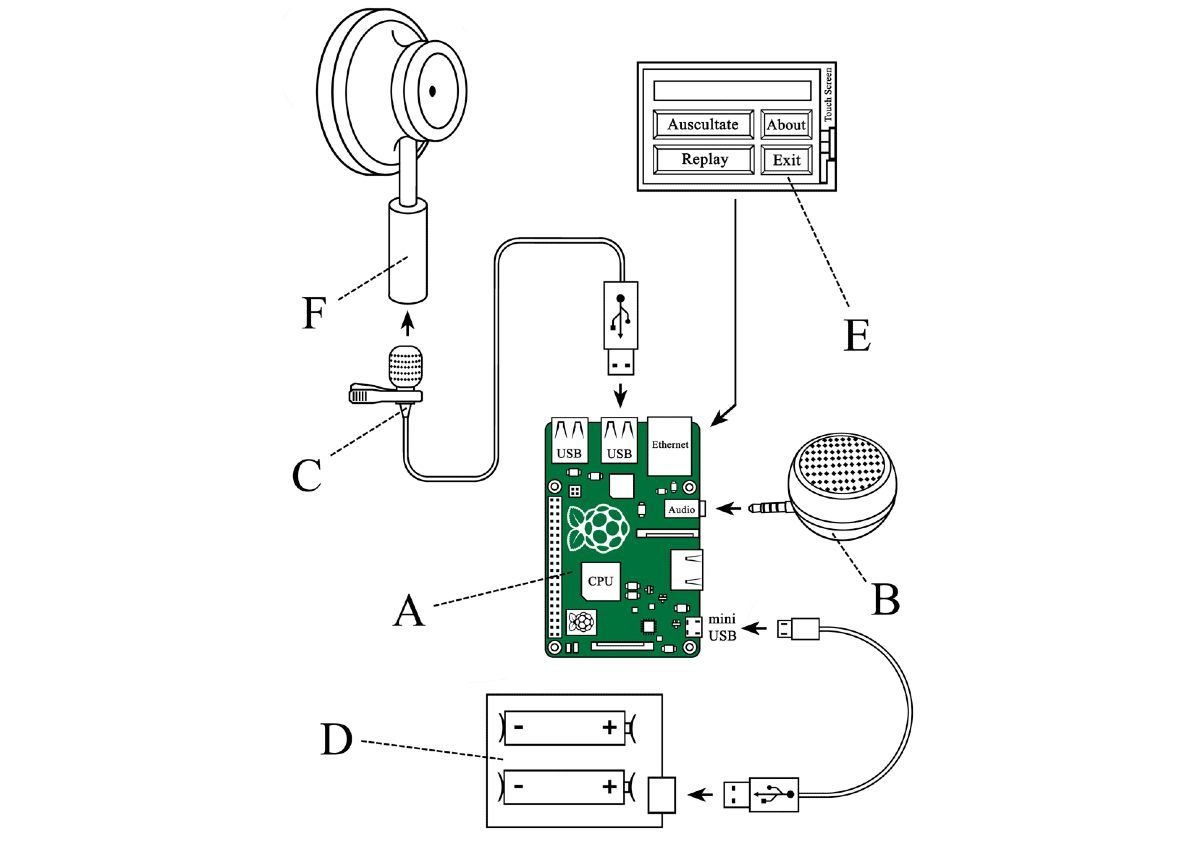
Schematic of the connections within the Auscul Pi system. (A) Raspberry Pi 3 Model B+. (B) Microspeaker. (C) USB collar microphone. (D) Uninterruptible power supply battery expansion board with two rechargeable batteries (18650). (E) 3.5-inch touch screen. (F) Chest piece from a conventional stethoscope. CPU: central processing unit.

We installed the operating system on the Raspberry Pi and initiated it [26], then connected the components mentioned in Table 1. Raspberry Pi can potentially use any type of sensor to record data and transfer it to the software program. We used an ordinary USB collar microphone as a transducer from the modified chest piece of a stethoscope to collect sound wave signals and transform them to electronic signals via USB port. Then, the Python-coded program (Auscul Pi Console) received the digital information, processed it, and sent it back to the microspeaker. Figure 3 illustrates the connections of each component and the Raspberry Pi computer.

**Figure 4:**
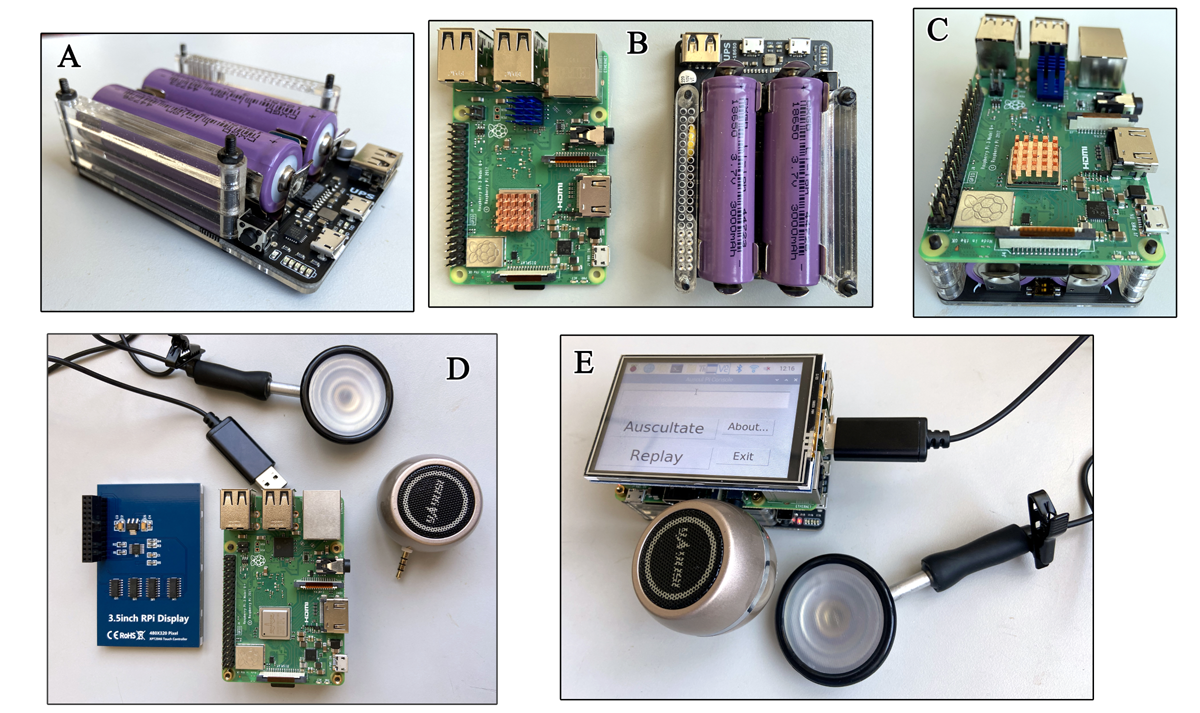
The Auscul Pi prototype. (A) The uninterruptible power supply. (B) The Raspberry Pi system (left) and uninterruptible power supply (right). (C) Combination of the Raspberry Pi and the power supply. (D) A microphone connected to the chest piece from a conventional stethoscope, 3.5-inch touch screen, Raspberry Pi with power supply, and microspeaker. (E) Fully assembled device containing the components in D.

Because the goal was to design an electronic stethoscope for use in a quarantine zone or ICU, it was necessary for its operation to be as simple as possible. We added a touch screen to initiate the auscultation and allow playback of the recorded sound. Figure 4 shows an image of the components of the Auscul Pi device.

#### Software

We used the Python programming language to code our software. Python is one of the most popular programming languages [27], not only because of its simplicity, excellent readability, and powerful functionality, but also because third-party professionals from diverse fields are using it to develop new packages and modules, which are uploaded to a shared repository called the Python package index [28]. Thus, Python is a “glue language” that can join different packages and modules together to construct code with desired functions.

**Figure 5:**
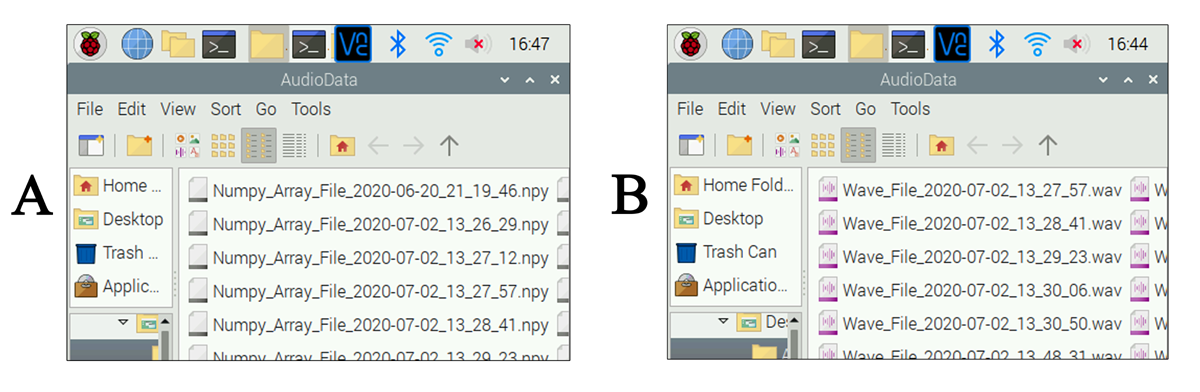
(A) Digital NumPy files (.npy) and (B) Waveform Audio File Format files (.wav) generated by Auscul Pi. The file names indicate the date and time of each measurement.

The PyAudio package is a third-party package developed for audio processing [29]. It can be downloaded from the GitHub repository [30] or installed by a Linux command (Multimedia Appendix 1). After importing PyAudio and other packages, we wrote our code. Our application program, Auscul Pi Console, was run on Raspberry Pi in a graphical user interface (GUI) using *tkinter* [31]. When the Auscul Pi Console runs, the auscultation sound can be played and heard via the microspeaker. The program generates a Waveform Audio File Format sound file (.wav) and digital NumPy array file (.npy) [32] bearing the date and time of the measurement (Figure 5).

Finally, we converted the Python code file *AusculPiConsole.py* to frozen binary code using PyInstaller [33] (Multimedia Appendix 2). This process enabled the program to be run on another Raspberry Pi simply by double-tapping the icon, without the need for a Python interpreter. The entire source code of the Auscul Pi Console is available at our GitHub repository [34] and in Multimedia Appendix 3, and it can be reused according to the terms of the Massachusetts Institute of Technology License.

**Figure 6:**
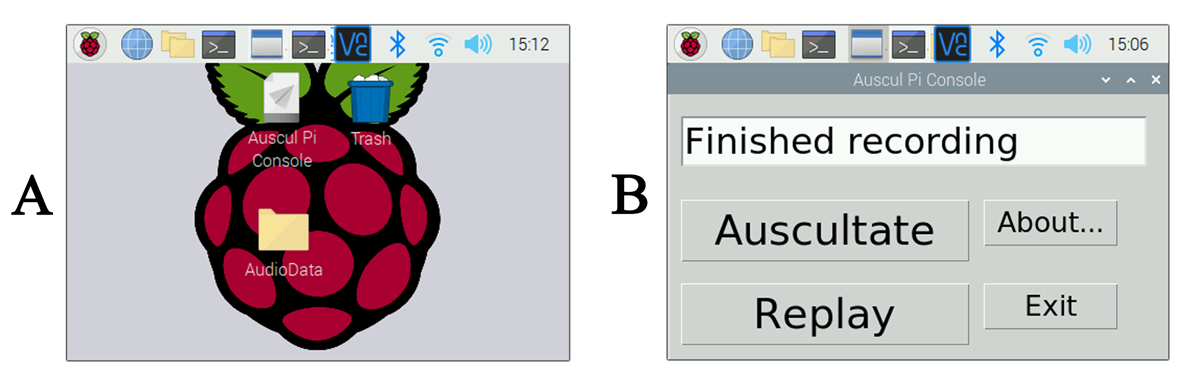
The graphical user interface on the touch screen of Auscul Pi. (A) The desktop display. The user can double-tap the Auscul Pi icon to run the program. The recorded data are stored in the AudioData folder. (B) The manipulation interface of the Auscul Pi Console after double-tapping the Auscul Pi Console icon. The user can control the auscultation and replay by tapping the “Auscultate” and “Replay” buttons, respectively.

This touch screen provides a GUI to activate the auscultation process. The “Auscultate” button is pushed, which initiates a 30-second recording and simultaneous broadcast of the auscultation. The last recorded auscultation can be played back by pressing the “Replay” button (Figure 6). The Auscul Pi Console program interface is user-friendly and can be operated interactively. All health care users in the present study were able to begin using the prototype quickly and were able to use it without touching the study participants.

#### Data Storage and Communication

The audio files and digital array files can be shared and transferred through a Wi-Fi signal for further study and analysis. For instance, we used Virtual Network Computing Viewer [35] or PuTTY terminal [36] to transfer the generated files to a personal computer. A phonocardiogram (PCG) records the occurrence of heart sounds in the cardiac cycle generated from the mechanical activity of the heart [37]. Our prototype includes a Python-coded parser program, which is also provided in our GitHub repository [38], that plots a PCG based on the auscultation data in the digital NumPy array file (.npy). PCG were generated from the Littmann stethoscope data using StethAssist software (3M).

### Clinical Study

After the installation of the hardware and the software, we applied this portable device in a pilot clinical study, which was approved by the Medical Ethical Committee of Shengjing Hospital of China Medical University (approval No. 2020PS525K); however, no application has been filed for commercial use to the regulatory agencies. Our pilot study (ChiCTR.org.cn Identifier ChiCTR2000033830) aimed to investigate the usability and advantages of Auscul Pi in comparison with the 3M Littmann 3200 electronic stethoscope. To assess the auscultation performance of heart sounds and respiratory sounds with Auscul Pi, we included eight patients with structural heart disease or heart failure and two healthy volunteers, who were examined face-to-face with the device in the clinic or inpatient department. None of the participants had been diagnosed with SARS-CoV-2 infection. Patients and volunteers gave written informed consent in this study.

Inclusion criteria were (1) patients with New York Heart Association class IV heart failure, from whom rale sounds, including moist crackles and wheezes, could be heard in lung auscultation; (2) patients with any type of structural heart disease, such as congenital heart disease or valvular heart disease, from which murmurs could be heard. Patients were excluded if they had weak heart sounds caused by pericardial effusion, pleural effusion, or pneumothorax. Two healthy volunteers were also included in the study.

We divided the participants into three groups according to the inclusion criteria: (1) the respiratory sound group (rale group) contained patients with heart failure; (2) the heart sound group (murmur group) contained patients with structural heart disease; and (3) the healthy group (normal respiratory sound and heart sound group) contained healthy volunteers. The auscultation procedure was different for each group. To collect respiratory sound from the patients with heart failure in the rale group, we auscultated their left and right lungs in the regions of the 7th to 9th intercostal space (7ICS to 9ICS) along the midaxillary line. We first performed auscultation with Auscul Pi by pressing the “Auscultate” button on the touch screen to initiate the 30-second recording and broadcast. We checked the respiratory sounds from the microspeaker without ear contact. During that time, we assessed whether we could clearly hear moist crackles or wheezes at the bedside. Then, we repeated the procedure using the Littmann stethoscope.

Before auscultation of patients with structural heart diseases in the murmur group, we checked the echocardiogram to locate the main origin of the murmur; then, we focused on the corresponding site for auscultation. For example, the echocardiogram of one patient with valvular heart disease showed mild mitral regurgitation. Therefore, we auscultated at the apex site, located around the 5ICS in the midclavicular line, to hear the loudest murmurs from the mitral valve. In one patient with congenital heart disease, the echocardiogram showed a ventricular septal defect. We auscultated in the 3rd intercostal space (3ICS) and 4th intercostal space (4ICS) to the left border of the sternum to hear the loudest murmurs. We listened carefully to the output from the microspeaker of the Auscul Pi to assess the presence and clearness of the murmurs. Next, we checked healthy volunteers with both stethoscopes to evaluate normal heart sounds and respiratory sounds.

After auscultation, we transferred the data from the two stethoscopes onto a personal computer via Wi-Fi (Auscul Pi) or Bluetooth (Littmann 3200). First, we listened to the sound files (.wav) from both stethoscopes and compared the respiratory and heart sounds and their quality. Second, we compared the PCGs generated from each stethoscope with each other and with the electrocardiograms (ECGs) showing the cardiac cycles. To quantify the consistency of the two PCGs, we evaluated the relationship of the waveforms between the Auscul Pi and the Littmann stethoscope by assessing whether they had similar simultaneous ups and downs in the waveform and whether they showed similar S1, S2, and murmur timings.

For the analysis of respiratory sound auscultation, we used the audio data collected from the patients with heart failure, and we listened to the audio file from our Auscul Pi stethoscope to evaluate the consistency with the results obtained from the 3M Littmann stethoscope. For the heart sound auscultation analysis, in addition to listening to the audio, three physicians (WZ, XZ, and SG) compared the PCGs plotted from the digital array files from each stethoscope for the morphologies of the waveforms. Furthermore, a Pearson correlation analysis was performed to assess the consistency of the results obtained with the two stethoscopes. We first processed the PCG data by extracting the wave amplitude values at every time point as a data series. Then, the correlation between the two data series was implemented to evaluate the peak and trough synchronizations of S1, S2, and murmurs using Python code, and we also provided the source code in our GitHub repository [39].

## Results

### Development

Auscul Pi is modular to enable construction of the entire device in a short time. We required 4 weeks to design, purchase, and assemble the hardware and code, debug, and optimize the software of Auscul Pi after we had the initial idea. Due to the size (10 cm × 6 cm × 5 cm) and light weight, the Auscul Pi can be carried with a single hand, while the other hand holds the chest piece of the stethoscope for auscultation. The standby time of the batteries was 2.5 hours during the auscultation examination, and the batteries could be fully recharged in 2 hours via the mini-USB port on the uninterruptible power supply extension board. We found that we could operate it conveniently and record the information precisely in our clinical practice when considering the aspects of ergonomics and information technology.

To make data-based decisions about the prototype design, we evaluated the performance of the Auscul Pi based on the seven dimensions mentioned in the Methods. We used a scoring system with 5 levels of satisfaction, each of which was each scored from 1-5, with 5 being the strongest score (most satisfactory) and 1 being the weakest score (least satisfactory). The evaluators were WZ, XZ, and SG, who gave the scores. All scores were weighted by importance to obtain the total scores. The total score of Auscul Pi was 104, which was higher than that of the conventional stethoscope (87) and the 3M Littmann stethoscope (82) (Table 2).

**Table 2:** Engineering design matrix showing the importance and satisfaction scores of various dimensions of the stethoscopes from 1-5 (1, weakest; 5, strongest).

Dimension	Importance	Satisfaction scores^a^		
		Conventional stethoscope	3M Littman digital stethoscope	Auscul Pi digital stethoscope
Size	2	5	4	3
Disinfectability	3	5	4	3
Ease of use	3	5	4	3
Affordability	3	5	3	4
Digitalization	3	1	3	5
Safety for patients	4	2	2	2
Safety for health professionals	4	1	1	4
Ability to detect auscultation sound	3	4	5	3
Usability in isolation in the intensive care unit	5	1	1	4
Total score	N/A^b^	87	82	104

^a^Satisfaction scores are weighted by importance.

^b^N/A: not applicable.

### Clinical Study

**Figure 7:**
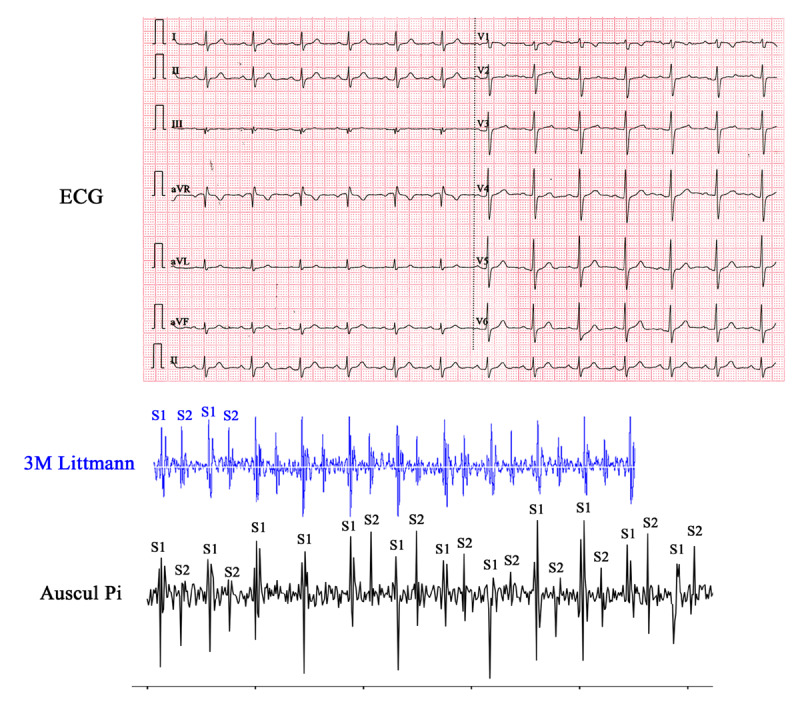
Electrocardiogram and phonocardiograms of healthy volunteer 9 generated by the 3M Littmann stethoscope and Auscul Pi, showing normal sinus rhythms and normal heart sounds. ECG, electrocardiogram: S1: first heart sound; S2: second heart sound.

This pilot study included eight patients and two healthy volunteers (Table 3). No patient was excluded from the study. First, we auscultated the two healthy volunteers to acquire normal heart sounds (Multimedia Appendix 4, Multimedia Appendix 5) and normal respiratory sounds (Multimedia Appendix 6, Multimedia Appendix 7). The audio of the respiratory and heart sounds obtained with Auscul Pi was clear and recognizable in both real-time play and the recorded archives. The digital NumPy array files of the corresponding sounds were used to plot the PCGs (Figure 7). The audio and PCGs generated by Auscul Pi were consistent with those obtained from the 3M Littmann stethoscope by the evaluations of the three physicians mentioned in the Methods section. To quantify the consistency of the two PCGs, we evaluated the relationship of the waveforms between the Auscul Pi and 3M Littmann stethoscopes by assessing whether they had similar peaks and valleys in the waveforms simultaneously, especially whether they had good synchronization of the first heart sound, second heart sound, and murmur timings. We firstly processed the PCG data by extracting the wave amplitude values of every time point. Then, we performed the Pearson correlation of the 2 data series, also with Python [39] (Multimedia Appendix 8). For the two healthy volunteers, the data for volunteer 9 had a correlation coefficient of 0.5570 (*P*<.001), and the correlation coefficient for volunteer 10 was 0.3245 (*P*<.001).

**Table 3:** Demographic characteristics of the patients and volunteers in the pilot study.

Number	Sex	Age (years)	Group	Diagnosis 1	Diagnosis 2	Auscultation site	Abnormalities
1	Male	64	Respiratory sound	Heart failure	Atrial fibrillation	7ICS^a^-9ICS along the midaxillary line	Wheezes
2	Male	79	Respiratory sound	Heart failure	Atrial fibrillation	7ICS-9ICS along the midaxillary line	Moist crackles
3	Male	43	Respiratory sound	Heart failure	Ischemic cardiomyopathy	7ICS-9ICS along the midaxillary line	Moist crackles
4	Male	66	Respiratory sound	Heart failure	Atrial fibrillation	7ICS-9ICS along the midaxillary line	Moist crackles
5	Male	68	Heart sound	Valvular heart disease	Mitral regurgitation	Apex (5ICS in the midclavicular line)	Mild holosystolic murmurs
6	Female	72	Heart sound	Valvular heart disease	Aortic stenosis	2ICS to the right border of the sternum	Mild holosystolic murmurs
7	Male	69	Heart sound	Valvular heart disease	Aortic stenosis	2ICS to the right border of the sternum	Mild holosystolic murmurs
8	Male	4	Heart sound	Congenital heart disease	Ventricular septal defect	3ICS and 4ICS to the left border of the sternum	Loud holosystolic murmurs
9	Male	40	Healthy	N/A^b^	N/A	Apex (5ICS in the midclavicular line)	None
10	Male	22	Healthy	N/A	N/A	Apex (5ICS in the midclavicular line)	None

^a^ICS: intercostal space.

^b^N/A: not applicable.

In the respiratory sound group (patients 1-4), all patients initially complained of dyspnea when presented at the hospital, and we heard rales in all patients. All these patients received anti-heart failure therapies. They all recovered and were discharged several days later. Patient 1 presented with atrial fibrillation and heart failure. At the beginning of the treatment period, we performed auscultation on this patient with both stethoscopes. We could hear clear wheezes in both the inspiratory and expiratory phases. During the examination, we checked the respiratory sounds simultaneously played by the microspeaker, from which the wheezes were clear and obvious. Then, we replayed the wheezing sounds in the computer when away from the patient, and the wheeze sound quality and recognizability were better than those obtained when we broadcast the sound during recording (Multimedia Appendix 9). The quality of the respiratory sounds was good for the other three patients in the group (patients 2-4) during playback of the recording.

For the patients in the heart sound group (patients 5-8), we examined and easily detected the murmurs at the corresponding auscultation sites of the culprit valves or defects. For example, patient 8, who was suffering from congenital heart disease, had two intraventricular septal defects. When we auscultated him at 3ICS and 4ICS to the left border of the sternum, a loud holosystolic murmur was clearly detected with Auscul Pi (Multimedia Appendix 10). The acoustic characteristics and timings of the murmurs were quite similar to the murmurs heard with the Littmann stethoscope (Multimedia Appendix 11). This patient underwent surgical ventricular septal repair. Postsurgery auscultation with the Auscul Pi (Multimedia Appendix 12) and Littmann stethoscope (Multimedia Appendix 13) stethoscopes showed that the murmurs had disappeared.

**Figure 8:**
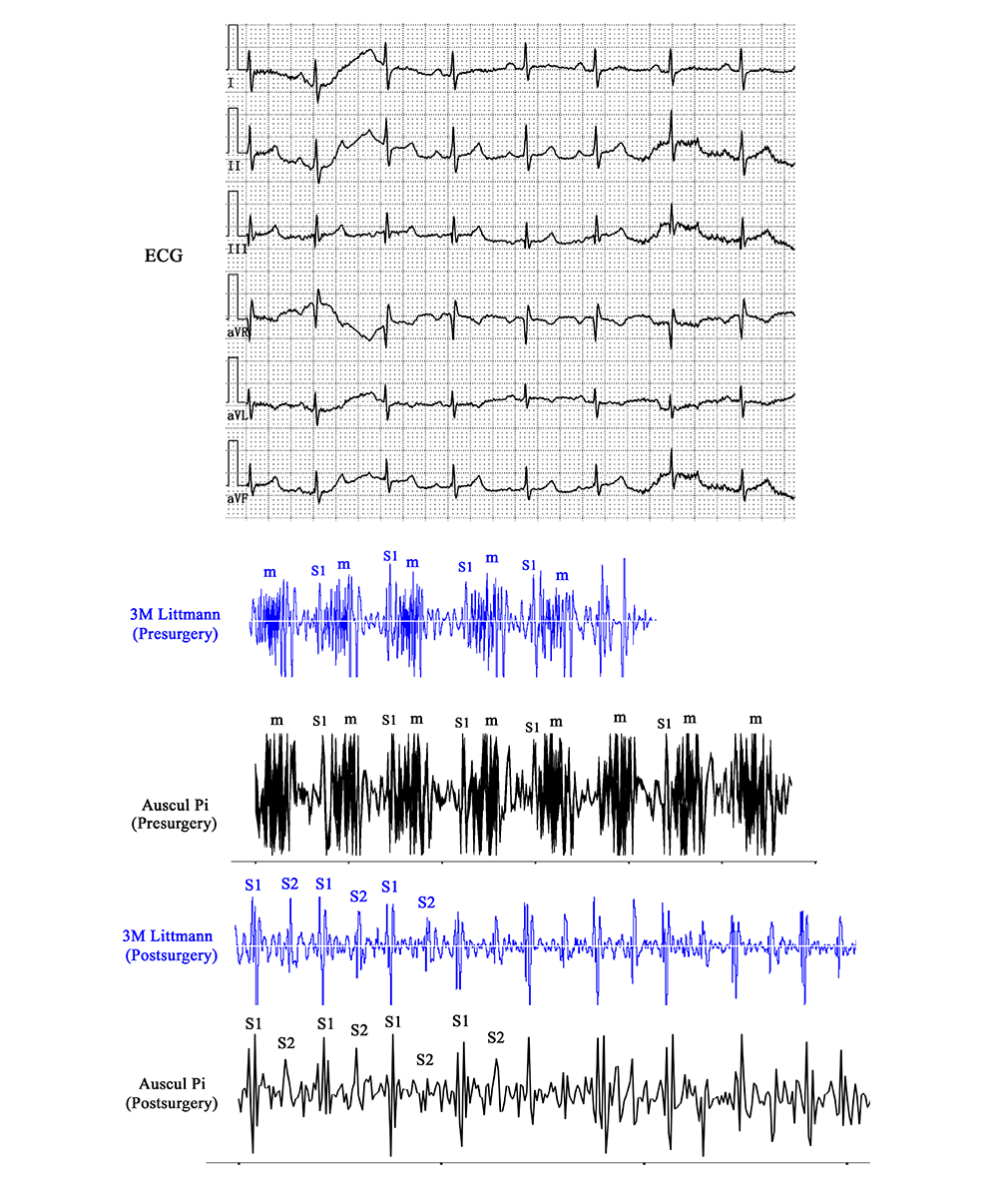
Electrocardiograms and phonocardiograms of patient 8, showing systolic murmurs before cardiac surgery to treat a ventricular septal defect but no murmurs after surgery. ECG: electrocardiogram; m: murmur; S1: first heart sound; S2: second heart sound.

The alignments of the PCGs with the ECGs showed good visual consistency between Auscul Pi and the 3M Littmann stethoscope (Figure 8). We also performed the same correlation analysis to evaluate the consistency of the data series extracted from the PCG NumPy data. The correlation coefficient of the Auscul Pi and 3M Littmann results before surgery was 0.3436 (*P*<.001), and the coefficient after surgery was 0.5138 (*P*<0.001). The correlation coefficients of the other 3 patients ranged from 0.3449-0.4797 (*P*<.001) (Table 4).

**Table 4:** Pearson correlation coefficients between phonograms obtained using the Auscul Pi and 3M Littmann stethoscopes (all *P* values <.001).

Patient or volunteer	Correlation coefficient
9	0.5570
10	0.3245
5	0.3449
6	0.4797
7	0.4134
8 (presurgery)	0.3436
8 (postsurgery)	0.5138

## Discussion

### Principal Results

In this work, we present the development of Auscul Pi, an innovative electronic stethoscope that we evaluated in a pilot study in patients with cardiovascular diseases. Our results show that Auscul Pi can be applied for the examination of cardiovascular diseases, as it clearly plays and records heart and respiratory sounds. The consistency between the results obtained by Auscul Pi and a typical electronic stethoscope was dependable based on qualitative analysis of the audio files and statistical analysis of the PCGs.

Our team found that Auscul Pi had several advantages over traditional stethoscopes in clinical practice. First, the contactless design of the stethoscope allowed no contact with the ears of the medical staff. The device can be used by physicians and nurses wearing a protective suit, eye protector, and face shield when auscultation is essential in clinical work, such as treating patients with COVID-19. The stethoscope can reproduce respiratory and heart sounds with the microspeaker, enabling nearby medical staff to hear the broadcast as well as if they were touching their ear to a stethoscope earpiece.

Second, the components are inexpensive, and component assembly and software installation are relatively easy; therefore, the do-it-yourself protocol can be followed by medical staff in a short time. The cost of the entire device is approximately US $94, of which $35 correspond to the Raspberry Pi; this is much lower than the price of a 3M Littman stethoscope. Therefore, the device is accessible to most medical facilities, hospitals, and emergency rooms worldwide. Additionally, Raspberry Pi is a popular project worldwide. It can be easily ordered on the web, and due to its small size, fast shipment is possible.

Third, in addition to its low cost, Raspberry Pi has versatility for application in many medical projects, ranging from assistance with medical imaging [40] to cervical cancer prevention [41], building computational microscopy [42], and ventilator buildup during the COVID-19 pandemic [42,43]. Moreover, Raspberry Pi uses Linux as a routine operating system, which is open-source, generic, and freely downloadable. Additionally, many software packages developed by third-party developers use Python, which is one of the most popular programming languages and works well with Raspberry Pi.

Fourth, the recorded respiratory and heart sound data can be stored in the Raspberry Pi, then transferred to a personal computer for further analysis if the hospital, emergency department, ICU, or ward has a Wi-Fi signal.

Finally, Auscul Pi can quantify and visualize auscultation: the system simultaneously records and broadcasts the signal, and the resulting computer files can be transferred using Wi-Fi for offline analysis. PCGs can also be plotted based on the NumPy array files for ease of visualization and analysis. These data can also be used in the future for research related to diagnosis and prognosis, such as use of machine learning algorithms [24,44]. The device may also have educational value as a teaching aid for medical students.

While treating patients with COVID-19 in Wuhan, we developed the Auscul Pi to solve the problems of auscultation. In a long-term perspective, this innovation may not be limited to the examination of patients with COVID-19, and it may be applied to other infectious diseases to reduce the risk of infection of medical workers. Although we do not envisage that Auscul Pi will become a commercial medical product in a large market, we believe it may inspire biomedical engineers, bioinformatics researchers, clinicians, and computer scientists to create low-cost engineering technologies to benefit patients who have been severely affected by COVID-19. Low-cost portable medical devices based on Raspberry Pi and the Python programming language may even become useful as tools for self-monitoring and assessment by patients with COVID-19 under quarantine [45], especially in low-resource areas.

### Limitations

The auscultation sounds recorded and broadcast by Auscul Pi inevitably contain some noises due to background and electricity, such as tiny click and pop sounds, that nevertheless do not cover up the main auscultation sounds. Future work should focus on filtering out much or all of this background noise. Our clinical research was a small pilot study to explore the feasibility of using the device in patients; however, we have planned a randomized clinical trial involving more medical professionals as device users and more patients with auscultatory abnormalities. We will also use questionnaires and unstructured interviews to ask the professionals about the usability and reliability of the device.

### Conclusions

A low-cost electronic stethoscope device, Auscul Pi, enables auscultation without ear contact. The device enables real-time broadcast of auscultation sounds and simultaneous digital data storage for offline analysis. Auscul Pi may enable accurate auscultation of patients with COVID-19 by medical workers wearing protective suits, thereby helping to minimize risk of infection.

## Appendix

Multimedia Appendix 1 PyAudio installation.

Multimedia Appendix 2 Conversion of the Python code to frozen binary code.

Multimedia Appendix 3 Source code of Auscul Pi Console.

Multimedia Appendix 4 Heart sounds of healthy volunteer 9 recorded with Auscul Pi.

Multimedia Appendix 5 Heart sounds of healthy volunteer 9 recorded with the 3M Littmann stethoscope.

Multimedia Appendix 6 Respiratory sounds of healthy volunteer 9 recorded with the Auscul Pi.

Multimedia Appendix 7 Respiratory sounds of healthy volunteer 9 recorded with the 3M Littmann stethoscope.

Multimedia Appendix 8 The source code of the phonocardiogram processing and the correlation analysis.

Multimedia Appendix 9 Respiratory sounds of patient 1 with heart failure patient recorded by Auscul Pi. Clear wheezes were heard.

Multimedia Appendix 10 Heart sounds of patient 8 with congenital heart disease (ventricular septal defect) before surgery recorded by Auscul Pi. Loud holosystolic murmurs can be heard.

Multimedia Appendix 11 Heart sounds of patient 8 before surgery by the 3M Littmann stethoscope. The murmurs can also be heard.

Multimedia Appendix 12 Heart sounds of patient 8 after surgery recorded by Auscul Pi. The murmurs have disappeared.

Multimedia Appendix 13 Heart sounds of patient 8 after surgery recorded by the 3M Littmann stethoscope. The murmurs have disappeared.

## Abbreviations

ECG: electrocardiogram
GUI: graphical user interface
ICS: intercostal space
ICU: intensive care unit
PCG: phonocardiogram
