# Supplementary material for: A Low-Cost, Ear-Contactless Electronic Stethoscope Powered by Raspberry Pi for Auscultation of Patients With COVID-19: Prototype Development and Feasibility Study
Source: JMIR Med Inform. 2021 Jan 19;9(1):e22753. doi: 10.2196/22753 (PMC7817256; doi:10.2196/22753)
Supplement: Multimedia Appendix 1 [file medinform_v9i1e22753_app1.pdf]

Multimedia Appendix 1: PyAudio Installation

We used PyAudio as an API (application programming interface) free of charge under the MIT License. We also referenced a piece of source code about PyAudio written by an engineer markjay4k (https://github.com/markjay4k/Audio-Spectrum-Analyzer-in-Python).

PyAudio can be downloaded from package manager 'apt' (a package manager is a tool for installing and uninstalling packages with Linux command):

```
$ sudo apt install python3-audio
```
