# Supplementary material for: A Low-Cost, Ear-Contactless Electronic Stethoscope Powered by Raspberry Pi for Auscultation of Patients With COVID-19: Prototype Development and Feasibility Study
Source: JMIR Med Inform. 2021 Jan 19;9(1):e22753. doi: 10.2196/22753 (PMC7817256; doi:10.2196/22753)
Supplement: Multimedia Appendix 2 [file medinform_v9i1e22753_app2.pdf]

Multimedia Appendix 2: Conversion of the Python Code to Frozen Binary Code

We converted the Python code file `AusculPiConsole.py` to frozen binary code with PyInstaller:

```
$ pyinstaller AusculPiConsole.py -w
```

A binary code file named `AusculPiConsole.desktop` was then generated in the corresponding folder.
