# Supplementary material for: A Low-Cost, Ear-Contactless Electronic Stethoscope Powered by Raspberry Pi for Auscultation of Patients With COVID-19: Prototype Development and Feasibility Study
Source: JMIR Med Inform. 2021 Jan 19;9(1):e22753. doi: 10.2196/22753 (PMC7817256; doi:10.2196/22753)
Supplement: Multimedia Appendix 3 [file medinform_v9i1e22753_app3.pdf]

Multimedia Appendix 3: Source Code of Auscul Pi Console

Available on GitHub: https://github.com/YangChuan80/AusculPi-Console/blob/master/AusculPiConsole.py

--- Code begins here: ------------

```
import pyaudio

import numpy as np

import wave

import os

import datetime

import tkinter as tk

from tkinter import messagebox



chunk = 512

samp_rate = 44100



form_1 = pyaudio.paInt16

chans = 1
```

```
record_secs = 30     #record time

dev_index = 2


def about():

    about_root=tk.Tk()


    w = 370 # width for the Tk root

    h = 230 # height for the Tk root


    # get screen width and height

    ws = about_root.winfo_screenwidth() # width of the screen

    hs = about_root.winfo_screenheight() # height of the screen


    # calculate x and y coordinates for the Tk root window

    x = (ws/2) - (w/2)

    y = (hs/2) - (h/2)


    # set the dimensions of the screen

    # and where it is placed
```

```
    about_root.geometry('%dx%d+%d+%d' % (w, h, x, y))
    about_root.title('About Auscul Pi Console')

    label_version=tk.Label(about_root,text='Auscul Pi Console Version 1.0', font=('tahoma', 9))
    label_version.place(x=90,y=20)

    label_copyright=tk.Label(about_root,text='Copyright (C) 2020', font=('tahoma', 9))
    label_copyright.place(x=125,y=50)

    label_author=tk.Label(about_root,text='Author: Chuan Yang', font=('tahoma', 9))
    label_author.place(x=125,y=80)

    label_institution=tk.Label(about_root,text='Shengjing Hospital of China Medical University', font=('tahoma', 9))
    label_institution.place(x=45,y=110)

    label_author=tk.Label(about_root,text='License: The MIT License (MIT)', font=('tahoma', 9))
```

```
    label_author.place(x=90,y=140)


    button_OK=tk.Button(about_root, width=15, text='OK',
command=about_root.destroy)
    button_OK.place(x=105, y=180)


    about_root.mainloop()


def replay():
    #plays the audio file
    os.system("aplay " + wav_output_filename)


def button_callback():
    global frames, frames_numpy, wav_output_filename,
np_output_filename


    pathBase = '//home//pi//Desktop//AudioData//'


    now = datetime.datetime.now()
    wav_output_filename = pathBase + 'Wave_File_' + str(now)[:10] +
now.strftime("_%H_%M_%S.wav")
```

```
    np_output_filename = pathBase + 'Numpy_Array_File_' +
str(now)[:10] + now.strftime("_%H_%M_%S")

    p = pyaudio.PyAudio()

    #setup audio input stream
    stream = p.open(format = form_1,
                    rate=samp_rate,
                    channels=chans,
                    input_device_index = dev_index,
                    input=True,
                    frames_per_buffer=chunk)

    # the code below is from the pyAudio library documentation
referenced below
    #output stream setup
    player = p.open(format = form_1,
                    rate=samp_rate,
                    channels=chans,
                    output=True,
```

```
                    frames_per_buffer=chunk)


    text_status.delete('1.0', tk.END)

    text_status.insert('1.0', "Broadcasting & Recording")

    print("Broadcasting & Recording")


    frames = []

    frames_numpy = []


    for ii in range(0,int((samp_rate/chunk)*record_secs)):

        data = stream.read(chunk,exception_on_overflow = False)

        frames.append(data)


        data_numpy = np.fromstring(data, dtype=np.int16)

        player.write(data_numpy, chunk)

        frames_numpy.append(data_numpy)


    text_status.delete('1.0', tk.END)

    text_status.insert('1.0', "Finished recording")

    print("Finished recording")
```

```
    stream.stop_stream()

    stream.close()

    p.terminate()


    #creates wave file with audio read in

    #Code is from the wave file audio tutorial as referenced below

    wavefile = wave.open(wav_output_filename,'wb')

    wavefile.setnchannels(chans)

    wavefile.setsampwidth(p.get_sample_size(form_1))

    wavefile.setframerate(samp_rate)

    wavefile.writeframes(b''.join(frames))

    wavefile.close()


    np.save(np_output_filename, frames_numpy)


root = tk.Tk()

root.geometry("{0}x{1}+0+0".format(root.winfo_screenwidth(),
root.winfo_screenheight()))

#root.attributes('-fullscreen', True)
```

```
root.title('Auscul Pi Console')


# Text Editor
text_status = tk.Text(root, width=20, height=1, font=('tahoma', 26),
bd=2, wrap='none')
text_status.place(x=10, y=20)


# Buttons
button_auscultate = tk.Button(root, text="Auscultate", width=10,
font=('tahoma', 30), command=button_callback)
button_auscultate.place(x=10, y=100)


button_replay = tk.Button(root, text="Replay", width=10,
font=('tahoma', 30), command=replay)
button_replay.place(x=10, y=180)


button_about = tk.Button(root, text="About...", width=6,
font=('tahoma', 20), command=about)
button_about.place(x=300, y=100)
```

```
button_exit = tk.Button(root, text="Exit", width=6, font=('tahoma',
20), command=root.destroy)
button_exit.place(x=300, y=180)


root.mainloop()
```

--- Code ends ------------
