# Supplementary material for: A Low-Cost, Ear-Contactless Electronic Stethoscope Powered by Raspberry Pi for Auscultation of Patients With COVID-19: Prototype Development and Feasibility Study
Source: JMIR Med Inform. 2021 Jan 19;9(1):e22753. doi: 10.2196/22753 (PMC7817256; doi:10.2196/22753)
Supplement: Multimedia Appendix 8 [file medinform_v9i1e22753_app8.pdf]

Multimedia Appendix 8: Source Code of the Phonocardiogram Processing Program

1. Phonocardiogram Plotting Program: SoundParser

Available on GitHub: https://github.com/YangChuan80/AusculPi-Console/blob/master/SoundParser.ipynb

--- Code begins here: ------------

```
from PIL import Image
import numpy as np
import pandas as pd
import matplotlib.pyplot as plt
get_ipython().run_line_magic('matplotlib', 'inline')

pathBase = 'MyFolder'

filename = 'Numpy_Array_File_2020-06-24_18_15_52.npy'
line = pathBase + filename

arr = np.load(line)
print(arr.shape)



fig = plt.figure()
s = fig.add_subplot(111)
s.plot(arr[0])

start = 1675
end = 2200

start_adj = int(start * 2583 / 3000)
end_adj = int(end * 2583 / 3000)
```

```
fig = plt.figure()
s = fig.add_subplot(111)
s.plot(arr[start_adj:end_adj,240], linewidth=0.6, color='black')

fig = plt.figure()
s = fig.add_subplot(111)
s.plot(arr[start_adj:end_adj,100], linewidth=0.6, color='black')

fig = plt.figure()
s = fig.add_subplot(111)
s.plot(arr[start_adj:end_adj,240], linewidth=0.5, color='black')

fig.savefig('Phonocardiograph_Output.png', dpi=400)
```

--- Code ends ------------

2. Source Code of the Correlation Analysis: WaveformExtraction

Available on GitHub: https://github.com/YangChuan80/AusculPi-Console/blob/master/WaveformExtraction-yc.ipynb

--- Code begins here: ------------

```
from PIL import Image
import numpy as np
import matplotlib.pyplot as plt
from scipy import stats
import math
get_ipython().run_line_magic('matplotlib', 'inline')

image = Image.open('3Ms.bmp')
image

x = image.size[0]
y = image.size[1]
```

```
print(x)
print(y)

matrix = []
points = []
integrated_density = 0

for i in range(x):
    matrix.append([])
    for j in range(y):
        matrix[i].append(image.getpixel((i,j)))
        #integrated_density += image.getpixel((i,j))[1]
        #points.append(image.getpixel((i,j))[1])

redMax = 0
xStore = 0
yStore = 0
for xAxis in range(x):
    for yAxis in range(y):
        currentPoint = matrix[xAxis][yAxis]
        if currentPoint[0] ==255 and currentPoint[1] < 10 and currentPoint[2] < 10:
            redMax = currentPoint[0]
            xStore = xAxis
            yStore = yAxis

print(xStore, yStore)

# ### Extract Blue Points
redline_pos = 51
absMax = 0
littmannArr = []
points_vertical = []
theOne = 0

for xAxis in range(x):
    for yAxis in range(y):
```

```
        currentPoint = matrix[xAxis][yAxis]
        # Pickup Blue points
        if currentPoint[2] == 255 and currentPoint[0] < 220 and currentPoint[1] < 220:
            points_vertical.append(yAxis)



    # Choose the largest amplitude
    for item in points_vertical:

        if abs(item-redline_pos) > absMax:
            absMax = abs(item-redline_pos)
            theOne = item
    littmannArr.append((theOne-redline_pos)*800)

    absMax = 0
    theOne = 0
    points_vertical = []

fig = plt.figure()
s = fig.add_subplot(111)
s.plot(littmannArr, linewidth=0.6, color='blue')


# # Ascul Pi Data
pathBase =
'C://Users//triti//OneDrive//Dowrun//Text//Manuscripts//Data//YangChuan//AusculPi//'
filename = 'Numpy_Array_File_2020-06-21_07_54_16.npy'
line = pathBase + filename
arr = np.load(line)
arr
arr.shape

fig = plt.figure()
s = fig.add_subplot(111)
s.plot(arr[0], linewidth=1.0, color='black')
```

```
fig = plt.figure()
s = fig.add_subplot(111)
s.plot(arr[:,100], linewidth=1.0, color='black')

start = 1830
end = 2350

start_adj = int(start * 2583 / 3000)
end_adj = int(end * 2583 / 3000)

fig = plt.figure()
s = fig.add_subplot(111)
s.plot(arr[start_adj:end_adj,460], linewidth=0.6, color='black')

fig = plt.figure()
s = fig.add_subplot(111)
s.plot(littmannArr, linewidth=0.6, color='blue')

asculArr = arr[start_adj:end_adj,460]



# ## Preprocess the two array
asculArr_processed = []
littmannArr_processed = []

for item in asculArr:
    asculArr_processed.append(abs(item))

for item in littmannArr:
    littmannArr_processed.append(abs(item))

fig = plt.figure()
s = fig.add_subplot(111)
s.plot(asculArr_processed, linewidth=0.6, color='black')
```

```
fig = plt.figure()
s = fig.add_subplot(111)
s.plot(littmannArr_processed, linewidth=0.6, color='blue')

fig = plt.figure()
s = fig.add_subplot(111)
s.plot(asculArr_processed[175:375], linewidth=1.0, color='black')

fig = plt.figure()
s = fig.add_subplot(111)
s.plot(littmannArr_processed[:200], linewidth=1.0, color='blue')

len(littmannArr)
len(asculArr)

# ### Coeffient
stats.pearsonr(asculArr_processed, littmannArr_processed)
stats.pearsonr(asculArr_processed[176:336], littmannArr_processed[:160])

# ### Figures of Comparison
fig = plt.figure()
s = fig.add_subplot(111)
s.plot(arr[start_adj:end_adj,460][176:336], linewidth=0.6, color='black')
fig.savefig('PCG_yc_asculPi_comparison.png', dpi=500)

fig = plt.figure()
s = fig.add_subplot(111)
s.plot(littmannArr[:160], linewidth=0.6, color='blue')
fig.savefig('PCG_yc_littmann_comparison.png', dpi=500)

# ### Fitness
stats.chisquare(asculArr_processed[174:334], littmannArr_processed[:160])

def cosCalculate(a, b):
    l = len(a)
    sumXY = 0
```

```
    sumRootXSquare = 0
    sumRootYSquare = 0

    for i in range(l):
        sumXY = sumXY + a[i]*b[i]
        sumRootXSquare = sumRootXSquare + math.sqrt(a[i]**2)
        sumRootYSquare = sumRootYSquare + math.sqrt(b[i]**2)
    cosValue = sumXY / (sumRootXSquare * sumRootYSquare)
    return cosValue

cosCalculate(asculArr_processed[175:335], littmannArr_processed[:160])
fig.savefig('Phonocardiograph_Output.png', dpi=400)
```

--- Code ends ------------
